# Sick leaves among healthy pregnant Croatian healthcare workers during the COVID-19 pandemic due to loopholes in the occupational safety system

**DOI:** 10.2478/aiht-2024-75-3851

**Published:** 2024-06-29

**Authors:** Tea Samardžić, Roko Žaja, Jelena Macan

**Affiliations:** PROTEGO Healthcare Institution, Koprivnica, Croatia; University of Zagreb School of Medicine, Andrija Štampar School of Public Health, Zagreb, Croatia; Institute for Medical Research and Occupational Health, Zagreb, Croatia

**Keywords:** biological hazards, occupational exposure, pregnancy, risk assessment, risk mitigation, work ability, biološke štetnosti, procjena rizika, profesionalna izloženost, radna sposobnost, smanjenje rizika, trudnoća

## Abstract

The aim of this study was to explore occupational safety in pregnant Croatian healthcare workers (HCWs) during the coronavirus disease 2019 (COVID-19) pandemic. To this end we composed an anonymous questionnaire that included pregnancy data, risk assessment and mitigation, and workplace intervention and distributed it to HCWs through social media of their groups and associations. The study includes a total of 173 respondents (71.1 % physicians, 19.7 % nurses, 9.2 % other HCWs) diagnosed with pregnancy in 2020 and 2021. Employers were notified about HCWs’ pregnancy at the eighth (IQR 7.0–11.0) week of pregnancy, which delayed workplace risk assessment and mitigation beyond the first trimester. Only 19.6 % of the participants had the risk assessed and mitigated, mostly on their own initiative (76.5 %). After notifying employers about pregnancy, 37.0 % of participants opted for temporary work incapacity (TWI) due to “pregnancy complications” despite healthy pregnancy, 16.8 % were granted a pregnant worker’s paid leave at the expense of the employer, while 5.8 % continued to work at the same workplace. Nurses used the TWI benefit more frequently than physicians (58.8 % vs 30.1 %, P=0.004). Our findings suggest that occupational safety of pregnant HCWs in Croatia lacks clear-cut and transparent strategies to protect pregnant HCWs, forcing them to misuse the healthcare system.

## Sažetak

Cilj ovog istraživanja bio je istražiti zaštitu na radu trudnih hrvatskih zdravstvenih radnica (ZR) tijekom pandemije koronovirusne bolesti 2019 (COVID-19). U tu smo svrhu sastavili anonimni upitnik koji je uključivao podatke o trudnoći, procjeni i smanjenju rizika te o intervencijama na radnom mjestu i distribuirali ga ZR putem društvenih medija njihovih grupa i udruženja. Istraživanje je obuhvatilo ukupno 173 sudionice (71,1 % liječnice, 19,7 % medicinske sestre, 9,2 % ostale ZR) kojima je dijagnosticirana trudnoća 2020. i 2021. Poslodavci su o trudnoći ZR obaviješteni u osmom (IQR 7,0–11,0) tjednu trudnoće, što je odgodilo procjenu i smanjenje rizika na radnom mjestu nakon prvog tromjesečja. Postupak procjene i smanjenja rizika proveden je u samo 19,6 % sudionica, uglavnom na njihovu inicijativu (76,5 %). Nakon što su poslodavca obavijestile o trudnoći, 37,0 % sudionica koristilo je privremenu nesposobnost za rad (PNR) zbog „komplikacija u trudnoći” unatoč zdravoj trudnoći, 16,8 % odobren je plaćeni dopust trudne radnice na teret poslodavca, dok je 5,8 % nastavilo raditi na istom radnom mjestu. Medicinske sestre koristile su PNR češće nego liječnice (58,8 % prema 30,1 %, P=0,004). Naši rezultati upućuju na nedostatak jasnih i transparentnih strategija zaštite trudnih ZR u Hrvatskoj, što ih prisiljava na zlouporabu zdravstvenog sustava.

It is well known that healthcare workers (HCWs) were one of the most exposed groups to the severe acute respiratory syndrome coronavirus 2 (SARS-CoV-2) infection during the coronavirus disease 2019 (COVID-19) pandemic due to the nature of their work and their role in containing the pandemic (1,2,3,4). Considering that pregnant women are more susceptible to infections (5) and develop severe clinical forms of the disease (6), several organisations and health and safety at work experts (1, 7,8,9,10) suggested that pregnant HCWs should stop working with patients with suspected or proven SARS-CoV-2 infection, but there is little information about pregnant HCWs and their challenges with exposure to SARS-CoV-2 at the workplace during the pandemic. Only a few studies (11, 12) reveal that pregnant HCWs’ feared for their and foetus’ health and highlighted the lack of specific directives or guidelines for pregnant HCWs. Croatia is no different in this respect, as exposure to biological hazards is not contraindicated in pregnant HCWs (13,14,15), even though the SARS-CoV-2 was classified as a new high risk biological hazard (category 3, “one that can cause serious illness in humans and poses a serious danger to workers”) (16).

As there were no prompt guidelines for specific risk assessment and mitigation in pregnant HCWs during the COVID-19 pandemic (internal communication), the aim of our study was to explore the issue.

## PARTICIPANTS AND METHODS

### Participants

This cross-sectional study included HCWs who were pregnant and actively working during the COVID-19 pandemic. Volunteers were invited to participate through several HCW groups on the Facebook^®^ by filling in an online questionnaire. They were also encouraged to share the questionnaire with their colleagues. The questionnaire was also distributed by e-mail to several HCW associations (Table 1).

**Table 1:** Social media groups and associations through which we approached HCWs with an invitation to participate in the survey

**Croatian HCW Facebook groups:**
(original Croatian title / English translation)
Mladi liječnici u PZZ – konzultacije / Young doctors in PHC – consultations
Specijalizanti / Residents
Zdravstveni djelatnici u sustavu socijalne skrbi / Health workers in the social care system
Grupa za potporu i kritike djelatnicima Hitne medicinske pomoći – HMP / Group for support and criticism of Emergency Medical Aid workers – EMA
Medicinske sestre/tehničari – Glas sestrinstva / Nurses – The Voice of Nursing
Inicijativa medicinskih sestara-medicinskih tehničara / Nurse Initiative

**Croatian HCW associations:**
Croatian Chamber of Nurses, Croatian Chamber of Pharmacists, Croatian Chamber of Physiotherapists, Croatian Chamber of Midwives, Croatian Chamber of Dental Medicine, Croatian Chamber of Health Workers – The Professional Class for Medical Laboratory Work, The Professional Class for Occupational Therapy and The Professional Class for Health Radiological-technological Work, Croatian Psychological Chamber, Association for Reality Therapy of the Republic of Croatia, Croatian Chamber of Psychotherapists, Croatian Speech Therapy Association, Croatian Doula Association

PHC – primary health care. Note: one HCW can be a member of several groups and organisations at the same time

Following the current ethical standards (17), participants were informed about the survey in the introductory part of the questionnaire. By completing the questionnaire, they agreed to participate and accepted that the data would be used only to identify issues related to health protection and safety at work for pregnant workers. Participation was voluntary and anonymous.

The responses of 244 HCWs were received between 29 September and 22 October 2022, but the study includes only those by HCWs diagnosed with pregnancy in 2020 and 2021 (N=173, 70.9 %), considering that those were the years when the pandemic suppression measures were the strictest. Among them, 49.7 % were pregnant in 2020 and 50.3 % in 2021. 71.1 % were physicians, 19.7 % nurses, and 9.2 % psychologists, speech therapists, dentists, pharmacists, laboratory workers, midwives, and caregivers. Their median age was 31.0 years (interquartile range, IQR 28.0–34.0).

### Questionnaire

The questionnaire was created by the authors using the Microsoft Forms^®^ (Microsoft Corp., Redmond, WA, USA) and contained 48 items combining multiple-choice, true-false, and open-ended questions. Apart from the demographic data (age, education, work), the items were divided into four domains: pregnancy data, risk assessment, risk mitigation, and interventions at workplace.

The pregnancy domain consisted of questions about the year of diagnosed pregnancy (2020/2021), age at pregnancy, week of pregnancy at diagnosis, and when the employer was notified about the pregnancy, including reasons for delayed notification.

The risk assessment domain included questions about how familiar they had been with the statutory risk assessment of their specific job (position), attitude toward occupational hazards for pregnancy, worker’s and foetus’s health, and awareness of exposure to biological hazards at workplace, especially to persons tested positive for SARS-CoV-2.

The risk mitigation domain was composed of questions about compliance to general and specific measures for the SARS-CoV-2 infection risk mitigation, in other words, whether they implemented these measures, and whether personal protective equipment and COVID-19 vaccines were made available to them.

The interventions at the workplace domain consisted of questions concerning employers’ and pregnant HCWs’ actions after the pregnancy had been diagnosed, including individual risk assessment, counselling with occupational health practitioner (OHP), adjustment of work tasks, relocation to a safer position, receiving a healthy pregnant worker’s paid leave, using a sick leave due to pregnancy complications, or staying at the same workplace until mandatory maternity leave, 45 to 28 days before the term (18).

Here we mostly address the pregnancy data and interventions at the workplace domains.

### Statistical analysis

The results were analysed with descriptive and inferential statistics using counts and percentages for categorical data and medians for continuous data. Differences between HCWs were analysed with the Mann-Whitney *U* test for continuous data and Fisher’s exact and Fisher-Freeman-Halton test for categorical data. All *P* values below 0.05 were considered significant. All statistics were run on IBM SPSS Statistics for Windows, version 25.0 (IBM Corp., Armonk, NY, USA).

## RESULTS AND DISCUSSION

The findings of this study should be viewed in light of the limitations pertinent to data collection through social media and self-reporting. The questionnaire’s strength, however, lies in its anonymity, thanks to which pregnant HCWs felt free to report having taken sick leave even if the pregnancy was normal, as they saw no other option to protect pregnancy.

Table 2 lists healthcare institutions where the study participants worked. The participants were diagnosed with pregnancy in the sixth (IQR 6.0–8.0) week and informed their employers in the eighth (IQR 7.0–11.0) week of pregnancy. More than a third (37.6 %) did not inform their employers about pregnancy immediately after being diagnosed. The reasons for the delay were as follows: the opinion that pregnancy did not affect their work ability (52.3 %), the opinion that their workplace did not have a harmful effect on their own and foetus’s health (33.8 %), the fear of getting fired or being mobbed at work (20.0 %). Such a delay entails postponing risk mitigation beyond the first trimester, the most vulnerable period of the foetus’s development (19,20,21), as the working status of pregnant HCWs remains unclear: do they continue to work until further notice, are they on a sick leave and on what basis, do they have to take paid leave?

**Table 2:** Healthcare institutions where study participants worked (N=173)

**Healthcare institution**	**N**	**%**
Hospitals	121	69.9
Health centres	19	11.0
Croatian Institute of Public Health	8	4.6
Croatian Institute of Emergency Medicine	7	4.0
Pharmacies	6	3.5
Other healthcare facilities and nursing homes	12	7.0

Delaying to notify the employer about pregnancy over fear of losing the job or getting bullied points to psycho-social risks at their workplace. This is in line with recent studies reporting that younger women HCWs were exposed to mobbing (22,23,24,25,26,27,28) and puts additional burden on the psycho-physical health of pregnant workers, besides the one posed by the pandemic (11, 12, 29,30,31).

After having notified the employer about pregnancy, 139 or 80.4 % of the participants did not receive individual risk assessment, and 34 or 19.6 % received it, mostly on their own initiative (26 of 34 participants) rather than on the initiative of the employers (eight participants). This suggests that most employers – healthcare institutions no less – did not recognise pregnant HCWs as workers in need of special attention. Only 19 of the 34 participants who did receive individual risk assessment received it within two weeks of notifying the employers, while 14 had to wait for more than two weeks. Risk assessment was made by OHPs (for 21 participants) – who not once visited participants’ workplaces – participants themselves (19 participants), occupational safety experts (for 16 participants), and colleagues working at the same workplace (for six participants).

Furthermore, while the COVID-19 pandemic brought some positive revisions to individual risk assessment in Slovenia (32), mainly involving both the employer and an OHP, no such revision took place in Croatia (internal communication by occupational medicine specialists and safety experts). No professional guidelines for risk assessment were provided and neither were the guidelines for health protection of pregnant HCWs during the pandemic. Only recently did the Department of Occupational Health (within the national public health institute) publish a flowchart detailing protective actions to be taken after notifying employer of pregnancy (33).

With the aim of defining exposure risks at workplace, experts from Denmark, the Netherlands, and the United Kingdom developed a COVID-19 job exposure matrix (JEM), which establishes four determinants of transmission risk (number of people, nature of contacts, contaminated workspaces, and indoor/outdoor location), two mitigation measures (social distancing and face covering), and two factors for precarious work (income insecurity and proportion of migrants) (34). Similar JEMs have also been developed by other experts (35, 36), as such tools can greatly help to identify occupational risks and to create risk reduction policies for future epidemic outbreaks, especially when it comes to vulnerable workers. According to the national ordinance on risk assessment (37), once informed about worker’s pregnancy, the employer should already have complete workplace risk assessment information ready to decide whether the pregnant worker can continue to work at the current workplace and what adjustments are necessary, if any. At this point, OHPs, whose participation in the assessment is currently not mandatory, should be involved to speed up the process following pregnancy notification actions in terms of worker’s protection and safety.

Table 3 summarises participants’ answers as to what actions followed when they notified the employer about pregnancy. 37.0 % claimed temporary work incapacity (TWI) due to pregnancy complications, even though their pregnancy was normal, while 16.8 % were granted a paid leave by the employer. We believe that these quite desperate actions are owed to a sore lack of systematic approach to individual risk assessment and mitigation for HCWs in Croatia. Coincidentally, the pandemic years 2020–2022 saw an increase in the share of diagnosed “high-risk pregnancies” (36.5–37.7 % of all pregnancies) over complications that require close monitoring compared to the five-year period before the pandemic (27.5–33.3 %, from 2015 to 2019) (38). Such diagnosis and recommendation of monitoring, issued by a primary healthcare gynaecologist, entails sick leave. It is possible that our participants also contributed to this increase. All this suggests that a more transparent system of pregnancy sick leave entitlement is required in Croatia as is a better control of its use.

**Table 3:** Self-reported actions taken by participating HCWs (N=173) after having notified their employer about pregnancy

**Actions taken after having notified the employer about pregnancy**	**Participants (%)**
TWI due to pregnancy complications despite a normal pregnancy	37.0
Continuation of work at the same place with adjustment to reduce the risk, until MML or until the TWI occurs due to pregnancy complications	18.5
Healthy pregnant worker’s paid leave at the expense of the employer due to the impossibility of reducing the risk or transferring to other jobs	16.8
Continuation of work at the same place with acceptable risk, until MML or until the TWI occurs due to pregnancy complications	15.0
Transferring to other jobs with lower risk until MML or until TWI occurs due to pregnancy complications	6.4
Continuation of work at the same place with unacceptable* risk, until MML or until the TWI occurs due to pregnancy complications	5.8
Termination of employment	0.6

* according to the opinion of the participants. MML – mandatory maternity leave (45 to 28 days before term); TWI – temporary work incapacity

One limitation of our study is that we could not ascertain the facts about risk assessment, risk factors at the workplace other than SARS-CoV-2, or work ability during pregnancy or other self-reported items in the questionnaire, which is why our findings should be interpreted with some reserve.

Since only 5.8 % of our participant reported to have continued to work throughout pregnancy at workplaces they considered high-risk, more research is needed to evaluate HCWs’ knowledge of occupational risks. If this knowledge is found to be insufficient, HCWs would benefit from retraining that would help to build a safer working environment.

Table 4 summarises differences in between physicians and nurses as to what actions followed when they notified the employer about pregnancy. The reasons for these differences should be sought in different job tasks. Nurses more often come into close contact with patients and are the first to respond to patients’ inquiries and emergencies (4). If the risks cannot be minimised or the nurses transferred to a safer position, the employer is required to provide paid leave (15). The nurses in our study reported this to have happened significantly less often than did physicians, so more than a half (20 of 34 nurses) resorted to TWI due to pregnancy complications.

**Table 4:** Differences between physicians and nurses in self-reported actions taken after having notified the employer about pregnancy

**Actions taken after notifying the employer about pregnancy**	**Physicians N=123 (%)**	**Nurses N=34 (%)**	**P**
Continuation of work at the same place with unacceptable* risk, until MML or until the TWI occurs due to pregnancy complications	5.7	5.9	1.000
Continuation of work at the same place with acceptable risk, until MML or until the TWI occurs due to pregnancy complications	**19.5**	**0.0**	**0.002**
Continuation of work at the same place with adjustment to reduce the risk, until MML or until the TWI occurs due to pregnancy complications	16.3	17.6	0.800
Transferring to other jobs with lower risk until MML or until TWI occurs due to pregnancy complications	5.7	11.8	0.254
TWI due to pregnancy complications despite normal pregnancy	**30.1**	**58.8**	**0.004**
Healthy pregnant worker’s paid leave at the expense of the employer due to inability to minimise the risk or transfer to a safer job	**22.0**	**5.9**	**0.043**
Termination of employment	0.7	0.0	1.000

* according to the opinion of the participants. MML – mandatory maternity leave (45 to 28 days before term). TWI – temporary work incapacity

## CONCLUSION

Occupational safety procedures for pregnant HCWs in Croatia lack a systematic and more transparent approach to protecting their health and pregnancy, which came under the spotlight during the COVID-19 pandemic. Employers did not fully recognise pregnant HCWs as a vulnerable group who need individual risk assessment and mitigation at the workplace. The systemic weaknesses included late notification of pregnancy, which delayed any protection of the most vulnerable first trimester of pregnancy, and unclear procedures as to risk assessment and mitigation for this specific group of HCWs. In the absence of clear employers’ prevention strategies, pregnant HCWs resorted to sick leave due to pregnancy complications, even though there were none.

A solution to this issue would be to include OHPs in risk assessment for pregnant HCWs from the start, so that the prevention and protection measures during pregnancy are known in advance to both the worker and the employer, and that the loopholes in the healthcare system are not misused. This means that OHPs and specialists in gynaecology and obstetrics should cooperate more in protecting the health of the pregnant worker and preserving their ability to work, not only in terms of exposure to biological hazards but other workplace risks as well. Employers should encourage healthy interpersonal relations at the workplace and implement measures to prevent psycho-social risks, so that HCWs feel safe to report pregnancy in time.
